# Niacin Increases Adiponectin and Decreases Adipose Tissue Inflammation in High Fat Diet-Fed Mice

**DOI:** 10.1371/journal.pone.0071285

**Published:** 2013-08-13

**Authors:** Desiree Wanders, Emily C. Graff, B. Douglas White, Robert L. Judd

**Affiliations:** 1 Department of Anatomy, Physiology and Pharmacology, College of Veterinary Medicine, Auburn University, Auburn, Alabama, United States of America; 2 Department of Pathobiology, College of Veterinary Medicine, Auburn University, Auburn, Alabama, United States of America; 3 Department of Nutrition, Dietetics, and Hospitality Management, College of Human Sciences, Auburn University, Auburn, Alabama, United States of America; University of Padova, Italy

## Abstract

**Aims:**

To determine the effects of niacin on adiponectin and markers of adipose tissue inflammation in a mouse model of obesity.

**Materials and Methods:**

Male C57BL/6 mice were placed on a control or high-fat diet (HFD) and were maintained on such diets for the duration of the study. After 6 weeks on the control or high fat diets, vehicle or niacin treatments were initiated and maintained for 5 weeks. Identical studies were conducted concurrently in HCA_2_^−/−^ (niacin receptor^−/−^) mice.

**Results:**

Niacin increased serum concentrations of the anti-inflammatory adipokine, adiponectin by 21% in HFD-fed wild-type mice, but had no effect on lean wild-type or lean or HFD-fed HCA_2_^−/−^ mice. Niacin increased adiponectin gene and protein expression in the HFD-fed wild-type mice only. The increases in adiponectin serum concentrations, gene and protein expression occurred independently of changes in expression of PPARγ C/EBPα or SREBP-1c (key transcription factors known to positively regulate adiponectin gene transcription) in the adipose tissue. Further, niacin had no effect on adipose tissue expression of ERp44, Ero1-Lα, or DsbA-L (key ER chaperones involved in adiponectin production and secretion). However, niacin treatment attenuated HFD-induced increases in adipose tissue gene expression of MCP-1 and IL-1β in the wild-type HFD-fed mice. Niacin also reduced the expression of the pro-inflammatory M1 macrophage marker CD11c in HFD-fed wild-type mice.

**Conclusions:**

Niacin treatment attenuates obesity-induced adipose tissue inflammation through increased adiponectin and anti-inflammatory cytokine expression and reduced pro-inflammatory cytokine expression in a niacin receptor-dependent manner.

## Introduction

The incidence of obesity in the U.S. has reached epidemic proportions within the last 30 years. Obesity is associated with an increased risk of metabolic and cardiovascular disease (CVD), with CVD being one of the leading causes of morbidity and mortality in the U.S. [1]. Exaggerated adipose tissue lipolysis and increased serum non-esterified fatty acids (NEFAs) are characteristic features of obesity that often lead to the development of atherogenic dyslipidemia. Atherogenic dyslipidemia is a cluster of metabolic abnormalities characterized by moderately elevated low-density lipoprotein cholesterol (LDL-C; 130–159 mg/dL) and triglycerides (TGs; >150 mg/dL), small LDL particles and low high-density lipoprotein cholesterol (HDL-C; <35 mg/dL) [2]. Niacin clinically utilized as a monotherapy is an effective pharmacological intervention for the treatment of atherogenic dyslipidemia due to its ability to reduce carotid intima media thickness, improve endothelial function, and reduce CVD morbidity and mortality, presumably by improving blood lipid and lipoprotein characteristics. Along with producing modest reduction in circulating very low-density lipoprotein (VLDL), LDL-C, TG, and lipoprotein(a) concentrations, niacin is an effective pharmacological tool for increasing HDL-C concentrations [3]–[6].

Improvements in blood lipid metabolism produced by niacin have largely been attributed to its well-documented effects on adipose tissue lipolysis and hepatic triglyceride synthesis [7]. Niacin inhibits lipolysis through activation of the niacin receptor (HCA_2_) in adipose tissue, which transiently inhibits cAMP/PKA and reduces triglyceride hydrolysis and serum NEFA concentrations [8]. However, it has recently been demonstrated that the effects of niacin on lipids is independent of the niacin receptor and the suppression of free fatty acids [9].

It is becoming increasingly clear that niacin possesses pleiotropic benefits in addition to its known therapeutic effects on lipid metabolism. Evidence from our group and others indicates that niacin dramatically increases serum concentrations of the adipokine, adiponectin, in obese men with metabolic syndrome [10], [11]. Additionally, a single dose of niacin given orally or through intraperitoneal injection acutely increases serum adiponectin concentrations in rats and mice within minutes, and this effect is dependent upon activation of the niacin receptor [12]. Others have demonstrated that niacin treatment results in increased adiponectin mRNA [13], [14]. A decrease in circulating adiponectin concentrations is one of the most promising biomarkers of metabolic syndrome and CVD. Adiponectin possesses insulin-sensitizing, anti-atherosclerotic, and anti-inflammatory properties; therefore, at least some of the health-related benefits of niacin may be attributed to increased serum adiponectin concentrations.

More recently, studies have demonstrated that niacin has anti-inflammatory effects in a number of tissues including kidney [15], lung [16], 3T3-L1 adipocytes [14], monocytes [17], retinal pigment epithelial cells [18], and vascular endothelial cells [19], [20]. Niacin administration has been shown to reduce MCP-1, TNF-α, and IL-6 expression and NF-κB activation in kidney and lung of rodent models [15], [16]. Additionally, treatment with niacin reduces TNF-α-induced increases in the gene expression and secretion of the proinflammatory chemokines MCP-1, fractalkine and RANTES, and increases adiponectin mRNA in 3T3-L1 adipocytes [14]. Niacin treatment suppresses the NF-κB signaling pathway, resulting in reduced secretion of the proinflammatory cytokines and chemokine TNF-α, IL-6 and MCP-1 in isolated human monocytes and retinal pigment epithelial cells [18], [21]. It has been demonstrated that niacin inhibits vascular inflammation by decreasing endothelial reactive oxygen species production and inflammatory cytokine production [22]. Systemically, niacin reduces C-reactive protein and TNF-α, while also reducing hepatic inflammation through reduction in hepatic macrophage content [23], [24]. Niacin also inhibits monocyte chemotaxis [14]. Many of these anti-inflammatory properties of niacin have been linked to activation of the niacin receptor [17], [18].

Obesity is associated with decreased plasma adiponectin concentrations and a chronic low-grade inflammation characterized by increased adipose tissue expression of pro-inflammatory chemokines and cytokines such as MCP-1 [25] and TNF-α [26], as well as infiltration of M1 (pro-inflammatory) macrophages [27], [28]. Therefore, the objective of the current investigation was to determine the effects of niacin administration on obesity-induced adipose tissue inflammation through alterations in cytokine profile and macrophage infiltration of the fat, with a special emphasis on the role of adiponectin.

## Materials and Methods

### Materials

Niacin (nicotinic acid) was purchased from Sigma-Aldrich (St. Louis, MO). Rabbit polyclonal adiponectin and GAPDH antibodies were from Abcam, Inc. (Cambridge, MA).

### Animal Studies

Thirty-two 3–4 week old male C57BL/6 mice were purchased from Charles River Laboratories (Wilmington, MA). Mice were either placed on a control diet (10% kcal as fat; n = 16) or a high fat diet (HFD; 60% kcal as fat; n = 16) obtained from Research Diets (New Brunswick, NJ) for 11 weeks. After six weeks on the control or high fat diets, half of the mice from each group received niacin (approximately 200 mg/kg/day) dissolved in drinking water or vehicle (water) for four weeks. Niacin concentrations were increased to approximately 360 mg/kg/day for the fifth week of treatment. After five weeks of vehicle or niacin treatments, mice were fasted overnight (12 h) and euthanized by decapitation. Whole blood was collected and processed to isolate serum and tissues were flash frozen in liquid nitrogen and stored at −80°C until analysis.

Parallel studies were conducted in male global HCA_2_^−/−^ (receptor for niacin) mice. Thirteen HCA_2_^−/−^ mice were placed on the control diet (7 received vehicle; 6 received niacin), and nine HCA_2_^−/−^ mice were placed on the high fat diet (4 received vehicle; 5 received niacin). Initial HCA_2_^−/−^ mice breeding pairs were a generous gift from Dr. Stefan Offermanns (Bad Nauheim, Germany). All animal studies were approved by the Auburn University Institutional Animal Care and Use Committee prior to initiation, and maximum care was taken to minimize animal suffering.

### Serum Analysis

Serum total adiponectin and insulin concentrations were measured using ELISA kits from Millipore (Temecula, CA). Serum high-molecular weight (HMW) adiponectin concentrations were measured using an ELISA kit from Alpco Diagnostics (Salem, NH). Serum glucose and triglyceride concentrations were measured using kits from Cayman Chemicals (Ann Arbor, MI). Serum NEFAs were measured using a kit from Wako Chemicals (Richmond, VA).

### Real-time PCR

RNA was isolated from epididymal white adipose tissue (EWAT) using Qiagen RNeasy Lipid Tissue Mini Kit (Valencia, CA). RNA (0.5 or 1 µg) was reverse transcribed into cDNA using iScript cDNA Synthesis Kit from Bio-Rad. PCR primers used in the real-time-PCR analysis are listed in **Table 1**. Analyses were performed on a Bio-Rad iCycler iQ thermocycler. Samples were analyzed in 30 µl reactions using SYBR Green PCR Master Mix (Bio-Rad). All expression levels were normalized to the corresponding 36B4 mRNA levels, and are analyzed using the 2^−ΔΔCT^ method. 36B4 levels were unchanged in response to HFD or niacin treatment.

**Table 1:** Primers used for real-time RT-PCR.

Gene	Forward Primer 5′-3′	Reverse Primer 5′-3′
Adiponectin	GGAGAGCCTGGAGAAGCC	ATGTGGTAAGAGAAGTAGTAGAGTC
PPARγ	TTATGGAGCCTAAGTTTGAGTTTG	AGCAGGTTGTCTTGGATGTC
C/EBPα	GTGGAGACGCAACAGAAGG	CAGCGACCCGAAACCATC
SREBP-1c	AACCTCATCCGCCACCTG	GTAGACAACAGCCGCATCC
ERp44	CAGTCCACGAGATTCAGAGTC	AGAAAGGAAGGCACAGTCATC
Ero1-Lα	TGGAGCCGTGGATGAGTC	CCTTGTAGCCTGTGTAGCG
DsbA-L	ATCACGGAGTATCAGAGCATTC	GCAACAGTGGTGGGTAGC
MCP-1	GCATCTGCCCTAAGGTCTTC	CACTGTCACACTGGTCACTC
CD68	AGGCTACAGGCTGCTCAG	GGGCTGGTAGGTTGATTGTC
CD11c	GGAGCAGGTGGCATTGTG	GAGCGATGTCCTGTCTTGAG
IL-1β	GCAGCAGCACATCAACAAG	GTTCATCTCGGAGCCTGTAG
TNF-α	CGTGGAACTGGCAGAAGAG	GTAGACAGAAGAGCGTGGTG
IL-6	AGCCAGAGTCCTTCAGAGAG	GATGGTCTTGGTCCTTAGCC
IL-10	GCAGTGGAGCAGGTGAAG	CGGAGAGAGGTACAAACGAG
Arginase-1	TTGGCTTGCTTCGGAACTC	GGAGGAGAAGGCGTTTGC
Ym1	GCCCACCAGGAAAGTACAC	CTTGAGCCACTGAGCCTTC
MRC-1	ATTGTGGAGCAGATGGAAGG	GTCGTAGTCAGTGGTGGTTC
36B4	CACTGCTGAACATGCTGAAC	CCACAGACAATGCCAGGAC

**PPARγ** Peroxisome proliferator-activated receptor γ, **C/EBP**α CCAAT/enhancer-binding protein α, **SREBP-1c** Sterol regulatory element-binding protein-1c, **ERp44** Endoplasmic reticulum protein 44, **Ero1-Lα** Endoplasmic oxidoreductin-1-like protein, **DsbA-L** Disulfide-bond A oxidoreductase-like protein, **MCP-1** monocyte chemoattractant protein-1, **CD** Cluster of differentiation, **IL-1β** Interleukin-1β, **TNF-α** Tumor necrosis factor-α, **IL-6** Interleukin-6, **IL-10** Interleukin-10, **MRC-1** Macrophage mannose receptor C type 1.

### Immunoblot Analysis

EWAT pads (∼100 mg) were homogenized with a handheld homogenizer in RIPA buffer [NP40 (1%), sodium deoxycholate (24.1 mM), SDS (0.05%), NaCl (0.81%), Tris (25 mM, pH 6.8), EDTA (1 mM), supplemented with protease and phosphatase inhibitors] and the protein fractions of the epididymal fat were isolated. A DC protein assay (Bio-Rad) was then conducted on the samples to determine the protein concentration for each sample. Proteins (9 µg) were separated by SDS–PAGE (10%) and transferred to PVDF membranes. Membranes were blocked in 5% non-fat dry milk for 1 hour and incubated with primary antibody for 2.75 hours. Membranes were washed with TBS/0.1% Tween-20 three times and incubated with secondary antibody for one hour, then washed with TBS/0.1% Tween-20 three times. Blots were developed using a Kodak XOMAT 1000A film processor (Rochester, NY).

### Statistical Analysis

Data were analyzed using a 2x2x2 factorial ANOVA and regression analysis was performed using JMP software (SAS Institute Inc., Cary, NC). Significance was set *a priori* at *P*<0.05. Graphs were generated using GraphPad Prism software version 4.0 (GraphPad Software, La Jolla, CA).

## Results

### Effects of HFD and Niacin on Metabolic Parameters in Mice

HFD increased body weight, epididymal fat pad weight, serum glucose and insulin concentrations in both WT and HCA_2_^−/−^ mice (**Table 2**). Niacin had no effect on epididymal fat pad weight, serum glucose or insulin concentrations (**Table 2**). Interestingly, niacin tended to increase serum glucose concentrations in lean WT and HCA_2_^−/−^ mice, although this did not reach statistical significance. Niacin is known to suppress the release of free fatty acids from the adipocyte, resulting in a transient reduction in circulating NEFAs [29], [30]. In the current investigation, niacin reduced serum NEFA concentrations in the WT mice on the control diet only, but surprisingly did not produce a reduction in serum triglycerides (**Table 2**).

**Table 2:** Effects of HFD and niacin on metabolic parameters in mice.

	Control DietVehicle Niacin		High Fat DietVehicle Niacin		Control DietVehicle Niacin		High Fat DietVehicle Niacin	
**Body** **Weight (g)**	32.1±1.1	33.3±1.1	45.1±0.8^a^	40.9±1.9	29.5±1.1	31.5±0.9	38.7±1.0^a^	38.1±2.5
**Epididymal Fat** **Pad** **Wt (g)**	1.1±0.1	1.2±0.1	2.0±0.2^a^	2.0±0.2	1.0±0.2	1.3±0.1	2.9±0.3^a^	2.5±0.2
**Glucose** **(mg/dl)**	149.5±9.8	192.9±16.5	249.2±15.8^a^	235.7±18.4	157.4±19.2	204.7±8.7	247.5±10.8^a^	239.7±23.4
**Insulin** **(ng/ml)**	0.78±0.3	0.81±0.1	3.5±0.5^a^	2.5±0.8	1.06±0.4	1.57±0.1	2.91±0.4^a^	2.90±0.8
**HOMA-IR**	0.73±0.29	0.94±0.17	5.42±0.96^a^	3.93±1.30	1.28±0.68	1.98±0.19	4.35±0.44^a^	4.61±1.41
**Triglycerides** **(mg/dl)**	77.4±5.3	83.4±2.6	88.4±4.7	99.9±6.4	68.3±2.4	89.2±6.5	80.4±2.0	89.4±3.0
**NEFAs** **(mMol)**	1.2±0.1	0.84±0.1*	0.82±0.0	0.75±0.1	0.83±0.07	0.85±0.08	0.56±0.02	0.67±0.05
**Adiponectin** **(µg/ml)**	14.4±1.0	14.4±0.6	14.1±0.7	17.1±0.8*	11.7±0.5	12.2±0.6	14.1±0.8	15.2±1.4
**HMW** **Adiponectin** **(µg/ml)**	4.2±0.5	3.5±0.4	4.6±0.4	6.3±1.0	3.0±0.3	3.7±0.3	5.2±0.3^a^	5.7±0.4

* P<0.05 Compared to mice of the same genotype and diet receiving vehicle (i.e. drug effect); ^a^P<0.05 Compared to mice of the same genotype on control diet receiving vehicle (i.e. HFD effect). Values are means ± SEM.

### Niacin Increases the Anti-inflammatory Adipokine, Adiponectin

We and others have previously demonstrated that niacin treatment increases serum adiponectin concentrations [10], [31]. Adiponectin is an adipokine secreted predominantly from the adipose tissue with anti-inflammatory, insulin-sensitizing, and cardioprotective effects. In the current investigation, we found that niacin treatment increased serum adiponectin concentrations in WT mice on the HFD by 21%, but had no effect in WT mice on the control diet (**Table 2**) or HCA_2_^−/−^ mice on control or HFD (**Table 2**). The increase in total adiponectin was partially explained by a tendency for niacin treatment to increase high-molecular weight adiponectin in the WT HFD-fed mice (4.6±0.4 vs. 6.3±1.0 µg/ml).

**Figure 1:**
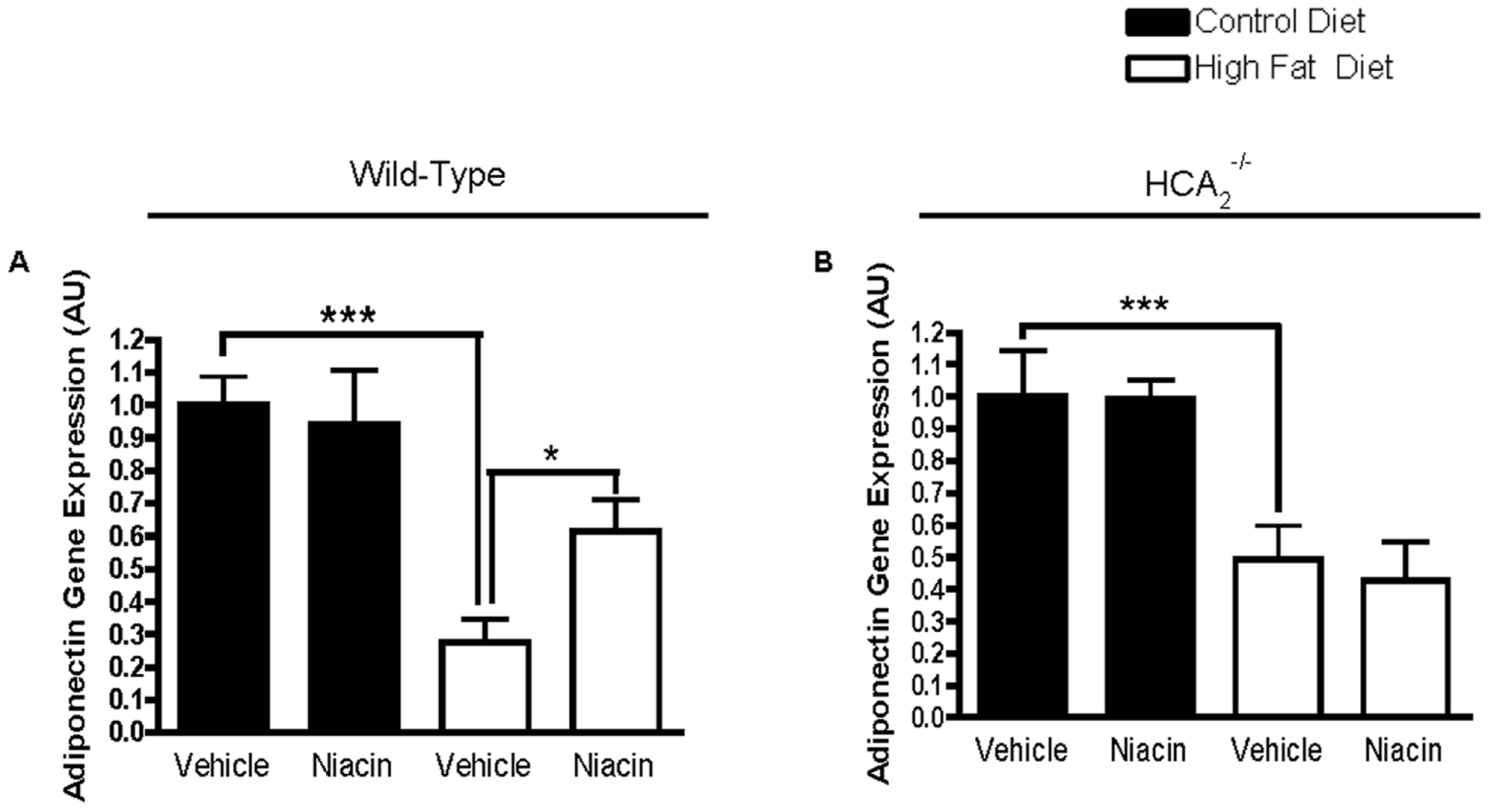
Effects of HFD and niacin on adiponectin gene expression. Adiponectin gene expression in EWAT of wild-type mice (A) or HCA_2_^−/−^ mice (B). Adiponectin values were normalized to 36B4. ^***^P<0.001 compared to mice on control diet vehicle; ^*^P<0.05 compared to mice on high fat diet receiving vehicle. Data are presented as mean ± SEM and are expressed as relative to the Control group set to one. **AU**, arbitrary units.

We then examined local production of adiponectin in the epididymal adipose tissue and found that both WT and HCA_2_^−/−^ mice on the HFD had significantly reduced adiponectin gene expression (**Figures 1A & B**). Niacin increased adiponectin gene expression in the WT mice on the HFD by 124%. (**Figures 1A & B**). The increase in adiponectin gene expression was not present in HCA_2_^−/−^ mice, which indicates that niacin increases adiponectin gene expression in a HCA_2_^−/−^dependent manner and that the increase in serum concentrations results from an increase in gene expression.

**Figure 2:**
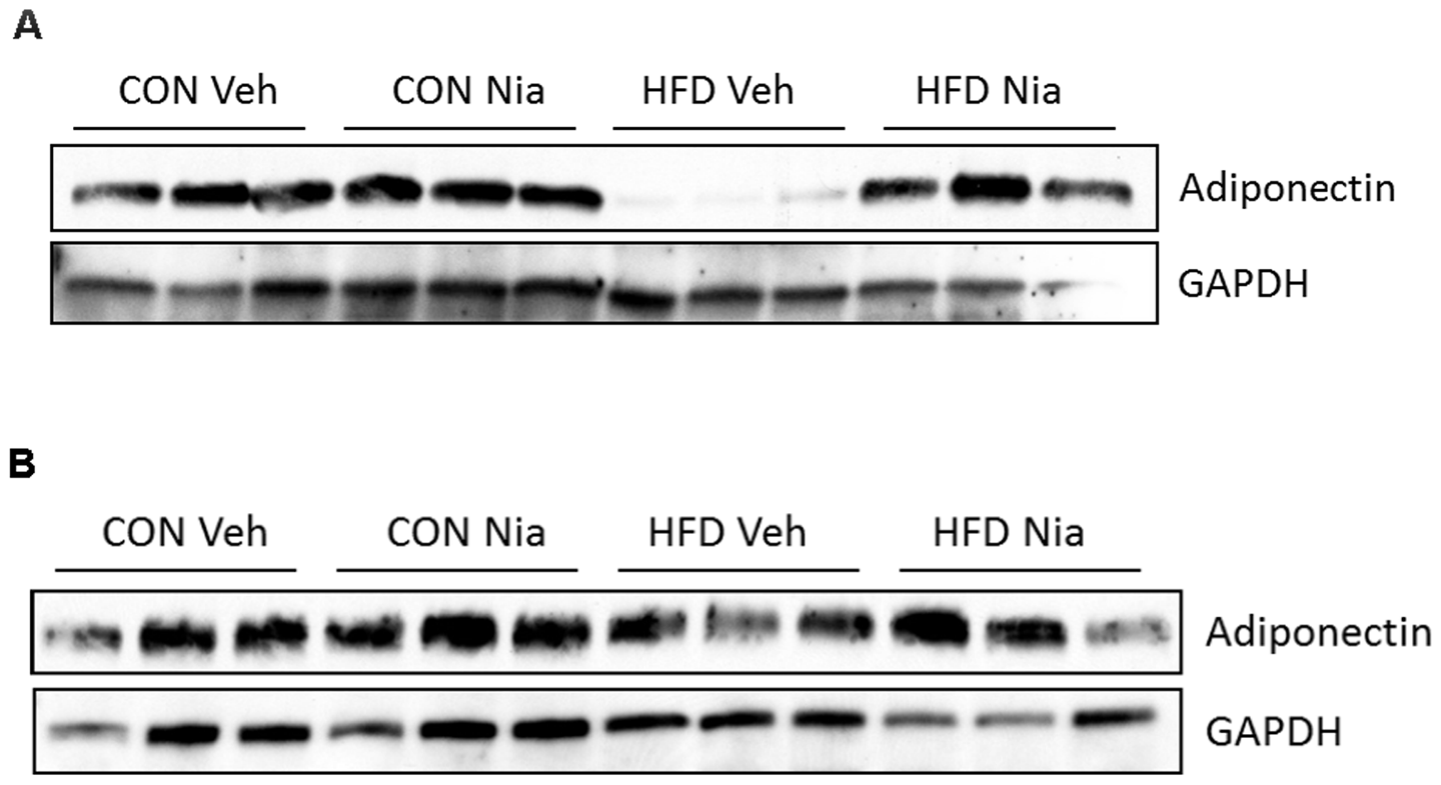
Effects of HFD and niacin on adiponectin protein expression. Adiponectin protein expression in EWAT of wild-type mice (A) or HCA_2_^−/−^ mice (B). 9 µg of protein were separated by SDS-PAGE and analyzed by immunoblot analysis.

In agreement with the adiponectin gene expression data, adiponectin protein expression in the epididymal fat pads was dramatically reduced in WT mice on the HFD (**Figure 2A**). Niacin increased adiponectin protein expression in the WT mice on the HFD (**Figure 2A**), but had no effect in mice on the control diet (**Figure 2A**). Interestingly, the HCA_2_^−/−^ mice appeared to be protected from the HFD-induced reduction in EWAT adiponectin protein expression (**Figure 2B**). Further, niacin had no effect on adiponectin protein expression in the EWAT of HCA_2_^−/−^ mice on control (**Figure 2B**) or HFD (**Figure 2B**).

### Niacin-mediated Increases in Adiponectin Occur Independently of Changes in Adipose Tissue Gene Expression of Transcription Factors or ER Chaperones Involved in Adiponectin Production

Cellular and secreted adiponectin levels can be regulated at the level of transcription. The adiponectin promoter contains binding sites for several transcription factors that have been shown to positively regulate adiponectin gene transcription including peroxisome proliferator-activated receptor γ (PPARγ) [32], CCAAT/enhancer-binding protein α (C/EBPα) [33], and sterol regulatory element-binding protein-1c (SREBP-1c) [34], [35]. Recent studies have demonstrated that niacin increases PPARγ activity and expression [36] and increases adipose tissue expression of C/EBPα [13]. Since we demonstrated that niacin increases adiponectin gene expression in the epididymal fat of HFD-fed WT mice, we examined the effects of niacin on these transcription factors.

**Figure 3:**
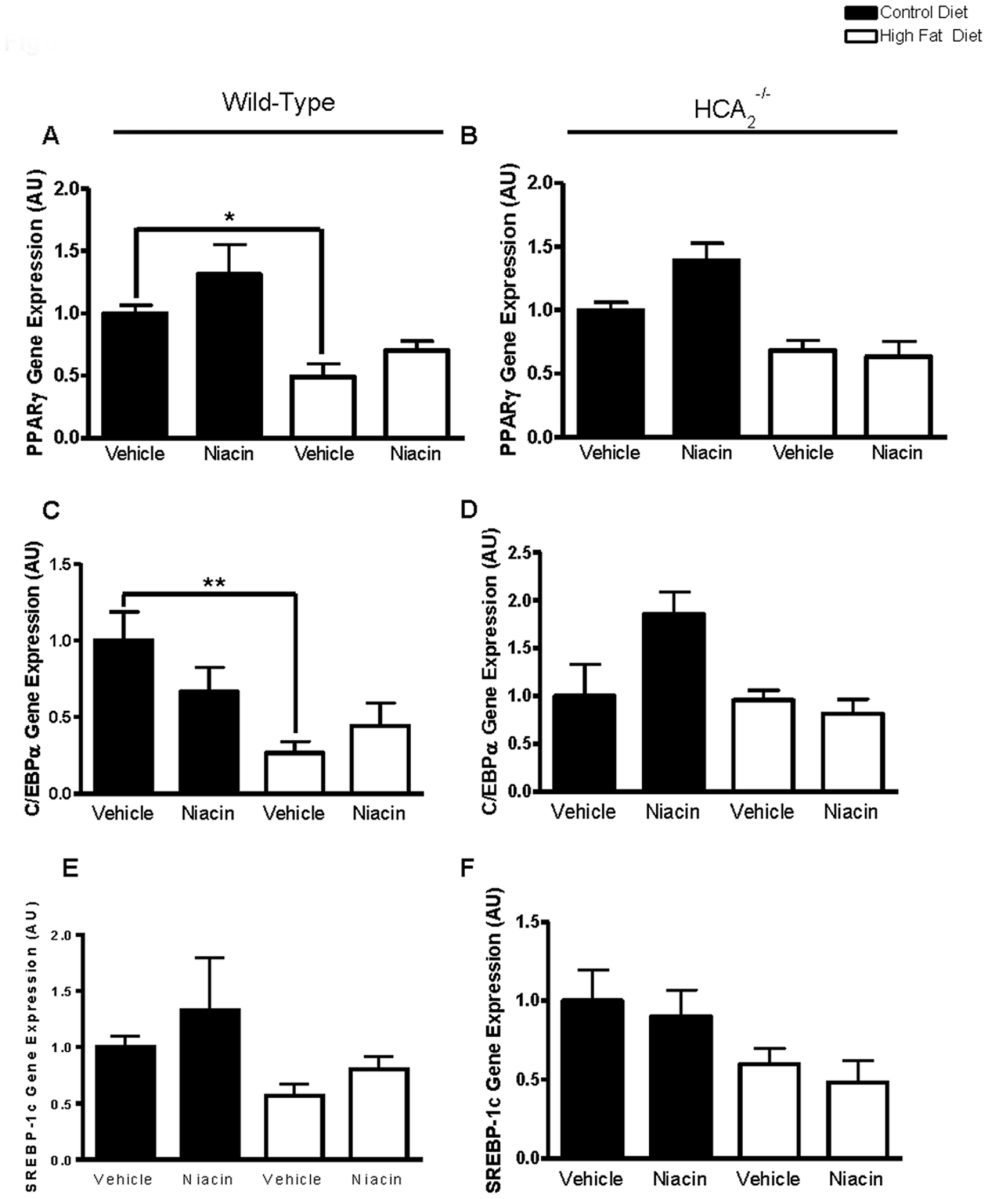
Effects of HFD and niacin on gene expression of transcription factors. PPARγ gene expression in EWAT of wild-type mice (A) or HCA_2_^−/−^ mice (B). C/EBPα gene expression in EWAT of wild-type mice (C) or HCA_2_^−/−^ mice (D). SREBP-1c gene expression in EWAT of wild-type mice (E) or HCA_2_^−/−^ mice (F). All values were normalized to 36B4. ^*^P<0.05, **P<0.01 compared to mice on control diet receiving vehicle. Data are presented as mean ± SEM and are expressed as relative to the Control group set to one. **AU**, arbitrary units.

PPARγ gene expression was reduced in the epididymal fat of WT mice on the HFD. However, niacin had no effect on PPARγ gene expression in the WT mice (**Figure 3A**). Unexpectedly, niacin increased PPARγ gene expression in the lean HCA_2_^−/−^ mice by 39% (**Figure 3B**). C/EBPα gene expression was significantly reduced in the EWAT of HFD-fed WT mice (**Figure 3C**), yet niacin had no effect on C/EBPα gene expression in any group of mice (**Figures 3C & D**). The adiponectin promoter is transactivated by SREBP-1c, with overexpression of SREBP-1c in 3T3-L1 adipocytes increasing adiponectin gene and protein expression [37]. We found that adipose tissue gene expression of SREBP-1c was unaffected by diet or niacin treatment in all groups of mice (**Figures 3E & F**).

**Figure 4:**
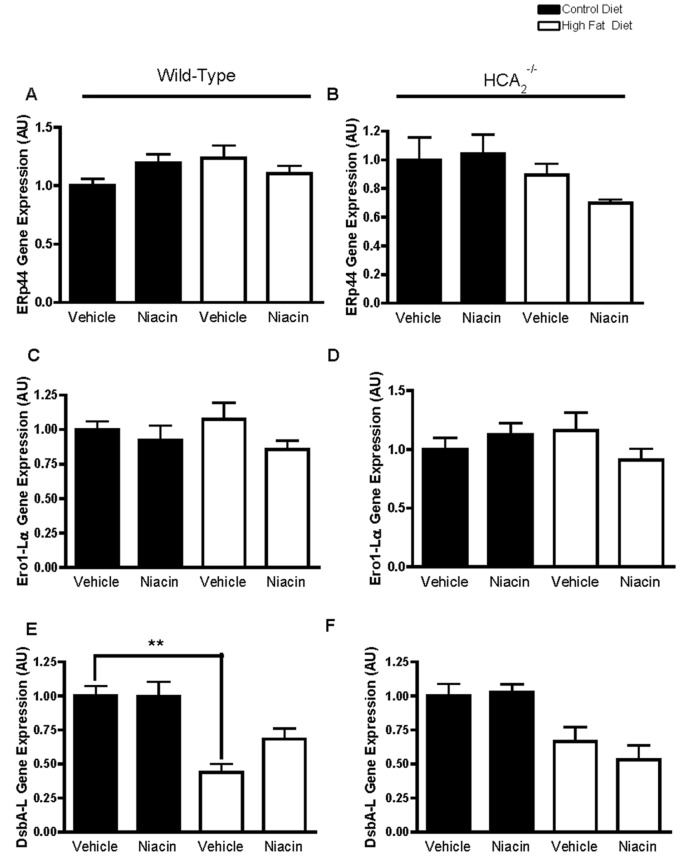
Effects of HFD and niacin on gene expression of ER chaperones. ERp44 gene expression in EWAT of wild-type mice (A) or HCA_2_^−/−^ mice (B), Ero1-Lα gene expression in EWAT of wild-type mice (C) or HCA_2_^−/−^ mice (D), and DsbA-L gene expression in EWAT of wild-type mice (E) or HCA_2_^−/−^ mice (F). All values were normalized to 36B4. **P<0.01. Data are presented as mean ± SEM and are expressed as relative to the Control group set to one. **AU**, arbitrary units.

The ER chaperones ERp44, Ero1-Lα, and DsbA-L are known to be involved in adiponectin production and secretion [38]. In order to address an additional mechanism by which niacin could be increasing serum adiponectin concentrations, we examined ERp44, Ero1-Lα, and DsbA-L gene expression in the epididymal adipose tissue. ERp44 and Ero1-Lα were not significantly affected by diet or niacin treatment in WT or HCA_2_^−/−^ mice (**Figure 4**). DsbA-L expression in the adipose tissue of wild-type mice was significantly reduced by HFD, and niacin tended to increase its expression in this group of mice, but not significantly (**Figure 4**).

### Niacin Attenuates HFD-induced Adipose Tissue Inflammation in a Receptor-dependent Manner

**Figure 5:**
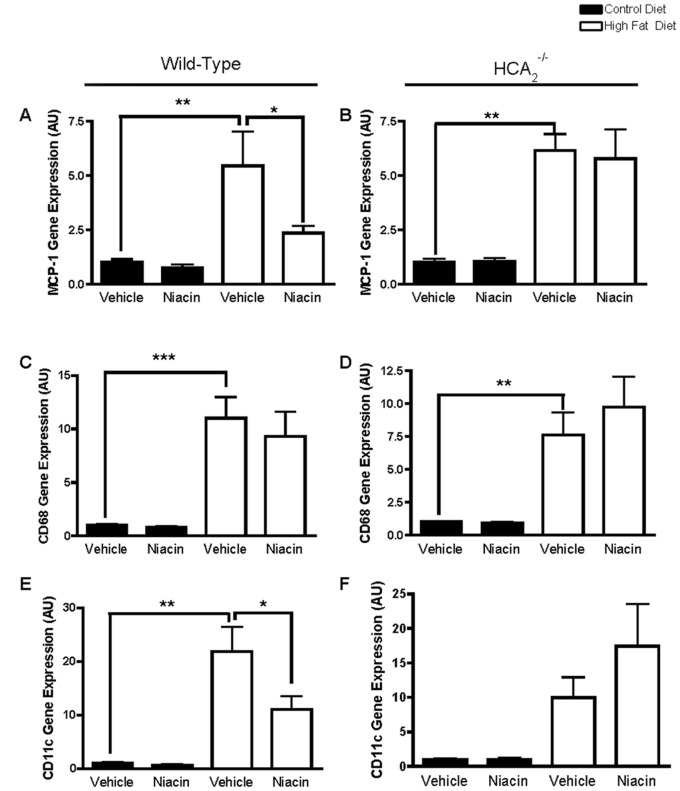
Effects of HFD and niacin on markers of adipose tissue inflammation. MCP-1 gene expression in EWAT of wild-type mice (A) or HCA_2_^−/−^ mice (B), CD68 gene expression in EWAT of wild-type mice (C) or HCA_2_^−/−^ mice (D), AND CD11c gene expression in EWAT of wild-type mice (E) or HCA_2_^−/−^ mice (F). All values were normalized to 36B4; *P<0.05, **P<0.01, ***P<0.001. Data are presented as mean ± SEM and are expressed as relative to the Control group set to one. **AU**, arbitrary units.

Anti-inflammatory effects of niacin have been demonstrated in many tissues including kidney [15], lung [16], adipocyte cell culture lines [14], monocytes [17], vascular endothelial cells [19], [20], and retinal pigment epithelial cells [18]. However, to date there have been no studies examining the anti-inflammatory properties of niacin in adipose tissue *in vivo*. Therefore, we examined the effects of niacin on adipose tissue inflammation, including expression of cytokines involved in inflammation and macrophage infiltration. As expected, HFD significantly increased MCP-1 gene expression in the adipose tissue of WT and HCA_2_^−/−^ mice (**Figures 5A & B**). Niacin reduced MCP-1 expression in the adipose tissue of HFD-fed WT mice, but not any other group of mice (**Figures 5A & B**), demonstrating novel anti-inflammatory effects of niacin in the adipose tissue. Since MCP-1 plays a major role in the recruitment of monocytes and macrophages to the adipose tissue, we examined the expression of the general monocyte and macrophage marker, CD68. As expected, HFD significantly increased CD68 expression in the adipose tissue, suggesting increased presence of macrophages in the EWAT (**Figures 5C & D**). However, niacin had no effect on CD68 expression. These results suggest that rather than altering the amount of macrophages present in the adipose tissue, niacin may be promoting a shift in macrophage polarization away from the M1 (pro-inflammatory), macrophage phenotype to the M2 (anti-inflammatory), phenotype. To address this, we examined adipose tissue expression of CD11c, a dendritic cell marker highly expressed on proinflammatory M1 macrophages, and not on anti-inflammatory M2 macrophages. HFD significantly increased adipose tissue expression of CD11c, indicating the presence of inflammatory macrophages, and niacin treatment significantly reduced CD11c expression in the fat of HFD-fed WT mice (**Figures 5E & F**).

**Figure 6:**
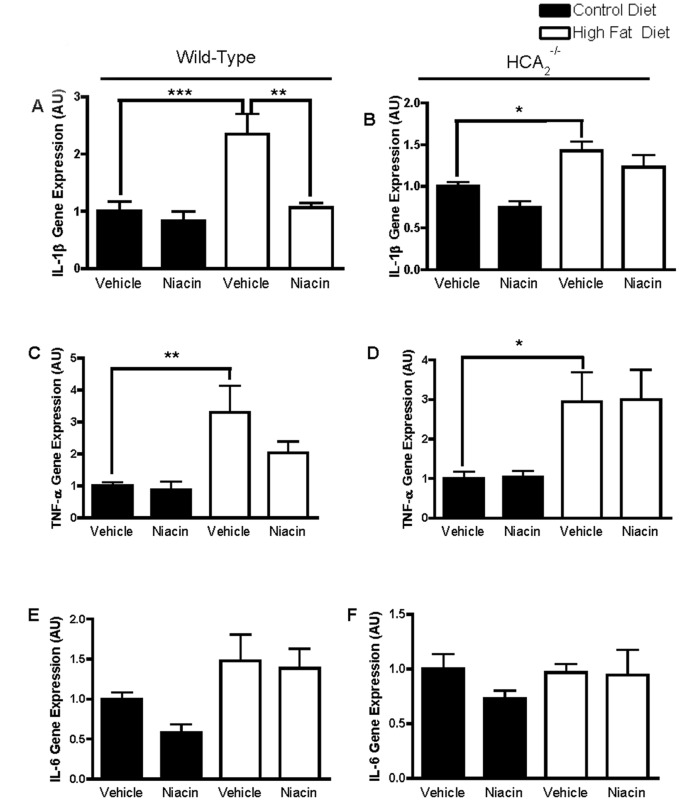
Effects of HFD and niacin on markers of adipose tissue inflammation. IL-1β gene expression in EWAT of wild-type mice (A) or HCA_2_^−/−^ mice (B), TNF-α gene expression in EWAT of wild-type mice (C) or HCA_2_^−/−^ mice (D), and IL-6 gene expression in EWAT of wild-type mice (E) or HCA_2_^−/−^ mice (F). All values were normalized to 36B4; *P<0.05, **P<0.01. Data are presented as mean ± SEM and are expressed as relative to the Control group set to one. **AU**, arbitrary units.

**Figure 7:**
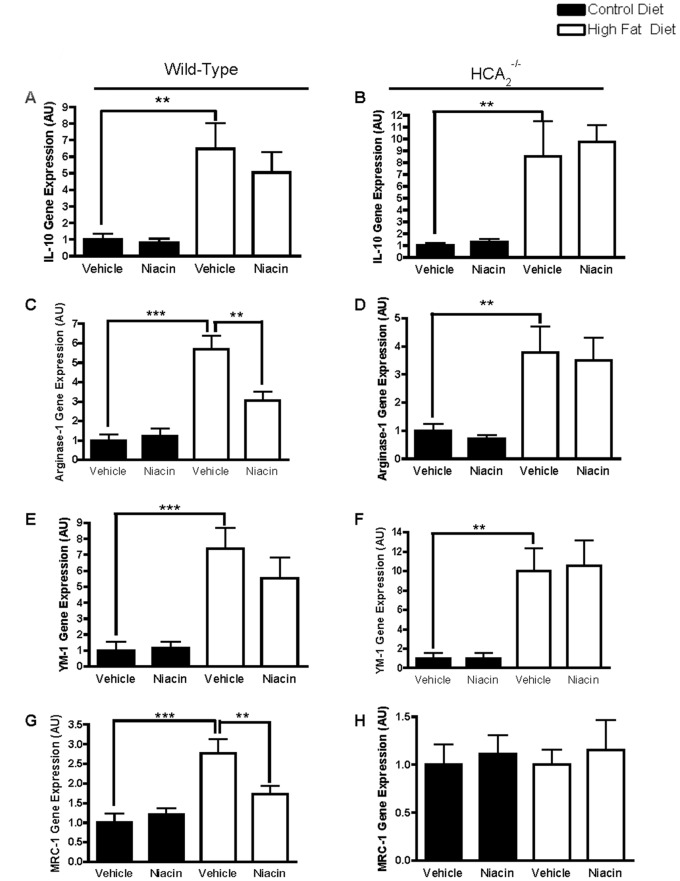
Effects of HFD and niacin on M2 macrophage markers. IL-10 gene expression in EWAT of wild-type mice (A) or HCA_2_^−/−^ mice (B), Arginase-1 gene expression in EWAT of wild-type mice (C) or HCA_2_^−/−^ mice (D), Ym1 gene expression in EWAT of wild-type mice (E) or HCA_2_^−/−^ mice (F), and MRC-1 gene expression in EWAT of wild-type mice (G) or HCA_2_^−/−^ mice (H). All values were normalized to 36B4; *P<0.05, **P<0.01. Data are presented as mean ± SEM and are expressed as relative to the Control group set to one. **AU**, arbitrary units.

Upon further examination of cytokine expression in the fat, we demonstrated that HFD increased gene expression of the pro-inflammatory cytokines IL-1β and TNF-α in the EWAT of both WT and HCA_2_^−/−^ mice. Niacin significantly reduced expression of IL-1β in the fat of HFD-fed WT mice (**Figures 6A & B**). This is important because IL-1β is linked to the pathogenesis of insulin resistance [39]. IL-1β is also highly expressed in inflammatory macrophages. The reduction in IL-1β could be indicative of niacin’s ability to reduce M1 macrophage presence in the adipose tissue. There was no effect of niacin on TNF-α expression in WT or HCA_2_^−/−^ mice (**Figures 6C & D**), and there was no effect of HFD or niacin on IL-6 expression (**Figures 6E & F**). IL-10 is an anti-inflammatory cytokine associated with macrophages of the M2 spectrum. Its expression was significantly increased in the EWAT of wild-type and HCA_2_^−/−^ mice on the HFD, and niacin had no effect on IL-10 expression in either group of mice (**Figures 7A & 7B**).

To determine if niacin’s anti-inflammatory effects in the adipose tissue are mediated through reduced presence of M1 and increased presence of M2 macrophages, we examined the expression of M2 macrophage markers in the fat. HFD increased adipose tissue expression of Arginase-1, Ym1 and MRC-1, genes that are highly expressed in M2 macrophages (**Figures 7C-H**), and unexpectedly, niacin treatment reduced Arginase-1 and MRC-1 gene expression in the EWAT of HFD-fed wild-type mice, but had no effect on Ym1. CD163, an M2 macrophage marker was unaffected by diet or niacin treatment in any group of mice (data not shown).

## Discussion

Niacin is now recognized to possess vascular anti-oxidant and anti-inflammatory properties that can contribute to improvements in cardiovascular outcomes, independent of alterations in blood lipid profile [40]. In fact, Ganji *et al.* demonstrated that niacin directly reduces endothelial reactive oxygen species production and subsequent LDL oxidation in cultured human aortic endothelial cells [22]. Others have shown that niacin reduces expression or release of pro-inflammatory mediators such as CRP, TNF-α and MCP-1 [14], [17], [23]. Lukasova *et al.* demonstrated that niacin inhibits atherosclerotic disease progression without altering total cholesterol, HDL-cholesterol or TG after 10 weeks in LDL receptor null mice [41]. The authors also demonstrated that niacin inhibits MCP-1-induced recruitment of macrophages to atherosclerotic plaques. We and others have demonstrated the ability of niacin to increase adipocyte production and circulating concentrations of the anti-inflammatory cytokine, adiponectin [10], [12], [14], [31]. These anti-inflammatory, lipid-independent effects of niacin may explain some of the health-related benefits of niacin in the face of unaltered blood lipid profile.

Adipocytes produce a number of adipokines that contribute both positively and negatively to the systemic inflammatory process. In adipose tissue of lean individuals, the adipocytes maintain a generally non-inflammatory cytokine profile. However, with obesity, the adipocyte hypertrophies to store excess triglyceride, and this hypertrophy is associated with dysregulation of adipokine secretion [42]. Hypertrophied adipocytes produce less of the anti-inflammatory adipokines including adiponectin and produce more pro-inflammatory cytokines such as TNF-α, contributing to the chronic inflammation seen with obesity [43], [44]. The mechanisms underlying HFD-induced reduction of adiponectin production include altered hormonal milieu [42], [45], [46], oxidative stress [47], and inflammation. Adipose tissue inflammation is characterized by increased expression of the inflammatory cytokines TNF-α and IL-6. TNF-α has inhibitory effects on adiponectin expression through suppressing its promoter activity [48], and IL-6 has also been shown to inhibit adiponectin expression in adipocytes [49]. Of the studies that demonstrated that chronic niacin administration increases serum adiponectin, only one elucidated possible mechanisms by which this occurs [13]. Linke *et al.* showed that six months of extended-release niacin increases serum adiponectin concentrations by 35%, and this was accompanied by a 4-fold increase in adiponectin mRNA expression in subcutaneous adipose tissue of humans with impaired glucose tolerance [13]. In addition, Linke *et al.* demonstrated that PPARγ and C/EBPα mRNA expression also increases 1.6- and 1.5-fold, respectively in the niacin-treated group. We showed no effect of niacin on PPARγ or C/EBPα gene expression in the epididymal fat of WT mice. However, this does not rule out the involvement of PPARγ in niacin’s ability to increase adiponectin. Examination of PPARγ activity or translocation to the nucleus may be of importance in order to further address the role of PPARγ in the mechanism of action of niacin. While we showed that the increased adiponectin mRNA and serum concentrations were not a result of increased PPARγ gene expression, others have shown that *in vitro*, niacin increases PPARγ mRNA expression [50], [51], induces nuclear expression of PPARγ protein and enhances PPARγ transcriptional activity [36].

In addition to the effects on adiponectin gene expression, PPARγ agonists can increase adiponectin production and/or secretion through alteration of ER chaperone expression [52]–[54]. ER chaperones including ERp44 and DsbA-L are important in adiponectin multimerization [38], while Ero-1Lα promotes the release of adiponectin from the adipocyte [54]. We therefore examined whether ER chaperone expression was altered by short term niacin administration and found that ERp44, Ero1-Lα and DsbA-L gene expression was unaffected by niacin treatment. Therefore, it does not appear that niacin increases adiponectin production through any of the mechanisms currently known to regulate adiponectin that we examined.

The ability of niacin to increase adiponectin may be just one of the multiple anti-inflammatory effects of niacin in the adipose tissue, or it may be a contributing factor to niacin’s direct anti-inflammatory properties. Adiponectin possesses anti-inflammatory properties including promoting macrophage polarization from an M1 towards and M2 phenotype [55]. Adiponectin treatment increases gene and protein expression of arginase-1, Mgl-1 and IL-10 and reduces expression of TNF-α and MCP-1 in isolated murine peritoneal macrophages [55]. Further, adiponectin suppresses the activity of NF-κB, a nuclear transcription factor involved in obesity-induced adipose tissue inflammation [56].

In the current investigation, we showed that niacin administration improved the inflammatory state of the adipose tissue through alterations in adipose tissue cytokine and macrophage content. Niacin attenuated HFD-induced increases in MCP-1, IL-1β and CD11c, genes associated with inflammatory M1 macrophages. We hypothesize that since adipose tissue expression of the general macrophage marker CD68 was unaltered by niacin in the face of reduced CD11c expression, that niacin treatment was promoting a shift in macrophage polarization away from the M1 towards the M2 phenotype. The macrophage profile of the adipose tissue is important, as increased M1 macrophage presence in the adipose tissue has been linked to obesity-associated insulin resistance [57].

M2 macrophages exist along a continuum and express different markers based on exposure to the microenvironment of the adipose tissue. In response to HFD feeding, while there is a clear increase in M1 macrophage markers, in many cases there are increases in M2 markers as well [28], [58], as we demonstrated with IL-10, arginase-1, MRC-1 and Ym1. Interestingly, niacin did not further increase the expression of M2 markers as we had hypothesized. Others have shown that HFD increases adipose tissue expression of the M2 markers arginase-1, Ym1, and IL-10 and that the insulin-sensitizing omega-3 fatty acid, DHA, further increases these M2 markers [59]. The DHA-mediated increases in arginase-1, Ym1, and IL-10 occurred in the stromal vascular fraction and not the adipocyte fraction of the adipose tissue [59]. While we also showed HFD-induced increases in the M2 markers arginase-1, Ym1, and IL-10, we found that niacin reduced arginase-1 expression and had no effect on adipose tissue expression of Ym1 and IL-10. Since many of the cytokines we examined are produced by both adipocytes and macrophages (MCP-1, TNF-α, IL-10, IL-1β), it will be important in the future to separate the stromal vascular fraction from the adipocyte fraction to determine the effects of niacin in each of these cell types. It’s also important to note that the two main sites of expression of the niacin receptor are on adipocytes and the immune cells, including macrophages, further demonstrating the importance of examining possible differential effects of niacin in these two cell types highly present in the adipose tissue. Initially, we were surprised that niacin reduced expression of markers associated with both pro-inflammatory M1 (CD11c, MCP-1, IL-1β) and anti-inflammatory M2 (arginase-1 and MRC-1) macrophages. Increased adipocyte production of MCP-1, free fatty acids, dead and dying adipocytes, hypoxia, oxidative stress, and ER stress have all been identified as factors that promote macrophage infiltration into adipose tissue [60]. Free fatty acids are known to drive both M1 and M2 macrophages into the adipose tissue. Increased local and systemic free fatty acids resulting from adipocyte lipolysis during weight loss induces the recruitment of macrophages to the adipose tissue [61]. Since niacin is a known inhibitor of adipocyte lipolysis, reduction in serum free fatty acids from the adipocyte could potentially inhibit the recruitment of M1 and M2 macrophages to adipose tissue.

The niacin receptor is primarily expressed in adipocytes and immune cells, including macrophages. Interestingly, niacin receptor expression on macrophages increases upon treatment with pro-inflammatory stimuli including TNF-α, interferon γ or LPS [62]. It is unknown whether the niacin receptor is expressed in the resident, anti-inflammatory M2 macrophages present in the adipose tissue of lean individuals. In the current investigation, we demonstrated anti-inflammatory effects of niacin in the HFD-fed WT mice only, which exhibit increased presence of pro-inflammatory M1 macrophages in the adipose tissue. It is possible that the reason we did not see anti-inflammatory effects of niacin in the lean mice is that niacin is exerting its anti-inflammatory effects through directly binding to its receptor on the adipocytes and M1 macrophages that are polarized to an inflammatory state, which are not present in adipose tissue from lean subjects.

Interestingly, beneficial effects of niacin on adipocyte biology have been demonstrated [13]. For example, adipocytes isolated from humans treated with niacin had reduced mean and maximal diameter and volume compared to control subjects [13]. Further, insulin sensitivity was also improved in these adipocytes. While we did not examine insulin sensitivity in our mice, there is evidence that niacin treatment improves insulin sensitivity in the adipocyte, and our findings suggest this could be due to reduced adipose tissue inflammation. However, clinically, it has been demonstrated that niacin increases blood glucose levels in diabetic and non-diabetic patients [3], so the effects of niacin on adipocyte insulin sensitivity likely do not contribute to improvements in whole-body insulin sensitivity.

In summary, the findings of the current investigation indicate that niacin reduces adipose tissue inflammation through reduced pro-inflammatory cytokine, chemokine and macrophage content and through increased serum and adipose tissue adiponectin concentrations. However, to fully elucidate the effects of niacin on adipose tissue inflammation, quantitative studies using fluorescence-activated cell sorting to determine quantity and type of macrophages present in the adipose tissue are necessary. The increases in adiponectin occurred independently of changes in expression of key transcription factors and ER chaperones involved in adiponectin production. These findings support the idea of pleiotropic effects of niacin to improve metabolic parameters in its target tissues, namely the adipose tissue. Further, all of these anti-inflammatory effects of niacin were lost in the niacin receptor knockout mice. This is consistent with previous reports that the niacin receptor is necessary to mediate anti-inflammatory properties of niacin [14], [17], [41]. In addition, it is important to note that the beneficial effects of niacin observed in HFD-fed mice were not present in lean WT mice. These findings suggest that the pleiotropic actions of niacin are greatest in the presence of obesity-induced metabolic dysfunction.
